# Prognostic Factors of Grade 2-3 Endo-Periodontal Lesions Treated Nonsurgically in Patients with Periodontitis: A Retrospective Case-Control Study

**DOI:** 10.1155/2020/1592910

**Published:** 2020-02-08

**Authors:** Xiaomiao Fan, Xiaoyu Xu, Shiwen Yu, Peicheng Liu, Chen Chen, Yaping Pan, Li Lin, Chen Li

**Affiliations:** ^1^School and Hospital of Stomatology, China Medical University, Liaoning Provincial Key Laboratory of Oral Disease, No. 117 Nanjing North St., Shenyang 110002, Liaoning Province, China; ^2^Department of Stomatology, Dalian Stomatology Hospital, Dalian, Liaoning, China

## Abstract

**Background:**

Endo-periodontal lesions are bacterial infectious diseases involving both the periodontal and pulp tissues with poor outcomes. It is hard for clinicians to predict their prognosis. The aim of this study is to investigate the factors affecting the prognosis of endo-periodontal lesions.

**Methods:**

A total of 140 teeth diagnosed with grade 2-3 endo-periodontal lesions in patients with periodontitis were recruited in this study. They were divided into high and low responder groups, according to the clinical symptoms and parameters of the teeth involved after nonsurgical treatment of both the endodontic and periodontal components. Clinical parameters and symptoms were compared before and after treatment, and gender, age, smoking, and all clinical parameters were compared between high and low responder groups using univariate analyses. Logistic regression was applied to evaluate the independent effects on endo-periodontal lesion prognosis.

**Results:**

Compared with the clinical parameters at baseline, the values of tooth mobility (TM), periapical index (PAI), and discomfort when chewing were decreased after endodontic therapy, and the values of periodontal probing depth (PD), clinical attachment level (CAL), sulcus bleeding index (SBI), TM, simplified oral hygiene index (OHI-S), full-mouth periodontitis severity, PAI, and discomfort when chewing were decreased after periodontal therapy. Univariate analysis revealed that smoking, PD, CAL, TM, PAI, clinical crown-root ratio (CR), full-mouth periodontitis severities, and the number of root canals were significantly different between the high and low responder groups (*P* < 0.05). The logistic regression analysis showed that smoking, PD, CAL, full-mouth periodontitis severities, and the number of root canals remained significantly associated with grade 2-3 endo-periodontal lesions in patients with periodontitis (*P* < 0.05). The logistic regression analysis showed that smoking, PD, CAL, full-mouth periodontitis severities, and the number of root canals remained significantly associated with grade 2-3 endo-periodontal lesions in patients with periodontitis (

**Conclusions:**

*and Practical Implications.* High PD and CAL, multirooted teeth, smoking, and serious full-mouth periodontitis indicated a poor prognosis for teeth with grade 2-3 endo-periodontal lesions.

## 1. Introduction

Endo-periodontal lesions have been characterized as bacterial infectious diseases that lead to extensive periodontal tissue damage and pulp inflammation or necrosis [1]. These lesions exist simultaneously in the periodontal and endodontic tissues of the same tooth [2]. Once endodontic and periodontal lesions are involved, especially in patients with periodontitis, the situation becomes more complex and requires extra considerations. Managing endo-periodontal lesions remains a challenge for clinicians.

With tooth development and root canal formation, three main avenues of communication between periodontal and endodontic tissues are created: the apical foramen, the lateral and accessory canals, and the dentinal tubules [3]. These special anatomical structures form an intimate continuum between the periodontal and endodontic tissues, through which pathological changes of either may lead to infection of the other. Bacteriological studies have reported similarities in the quantity and structure of the bacterial flora associated with endodontic and periodontal lesions; these findings indicate the communication between the periodontal and endodontic tissues [4–6].

Managing endo-periodontal lesions involves treating both endodontic and periodontal components, including initial periodontal therapy and root canal treatment. Some cases require periodontal and/or apical surgeries [3, 7–9], or even extraction because of poor prognosis. Clinically, when a tooth is diagnosed as having endo-periodontal lesions, the correct assessment of the prognosis of the involved tooth is of great relevance for clinicians in adopting a reasonable treatment plan [10–12]. Currently, clinicians usually make preliminary judgements regarding endo-periodontal lesion prognosis based on their own practical experience, which is insufficient and may lead to unnecessary treatment for some hopeless teeth, thus wasting time and money. Therefore, clinicians should determine whether an involved tooth will be maintained or not at baseline.

In 2018, the American Academy of Periodontology (AAP) published a new classification for endo-periodontal lesions. Grade 2-3 endo-periodontal lesions in patients with periodontitis are types of endo-periodontal lesions with a wide, deep periodontal pocket in 1 tooth surface or deep periodontal pockets in >1 tooth surface [10]. It is a disease with multiple etiologies, and the prognostic factors are also complicated and may include the severities of periodontal and endodontic lesions and the medical conditions of the patients. Which factors affect the prognosis of endo-periodontal lesions remains unclear. There have been few well-designed studies for these kinds of endo-periodontal lesions, and none of the relevant studies used multivariable models. Therefore, we conducted a retrospective case-control study using a multivariable model to investigate the factors for the prognosis of teeth with grade 2-3 endo-periodontal lesions in patients with periodontitis treated nonsurgically and to provide a reference for clinicians to make decisions for teeth involved in endo-periodontal lesions.

## 2. Materials and Methods

### 2.1. Case Selection

The study was registered on the World Health Organization International Clinical Trials Registry Platform (ChiCTR1800017541) and was conducted in conformity with the Declaration of Helsinki [13]. Clinical databases containing records on patients diagnosed with periodontitis and whose teeth were diagnosed with endo-periodontal lesions were searched from December 2016 to December 2018 at the Department of Periodontics, School and Hospital of Stomatology, China Medical University. The ethics committee of the School and Hospital of Stomatology, China Medical University, approved the study in 2014 under Number 12. The study's sample size was calculated using statistical software (Power Analysis and Sample Size, Version 11, Kaysville, Utah, USA) with a test power of 0.8 (1-*β*), an alpha probability of 0.05, an OR value 2.7, a sample allocation ratio of 1.0, a control group proportion of 0.35, and a case-control ratio of 1 : 1. The OR and control group proportion were based on the results of a preliminary experiment, and we chose smoking (OR = 2.7 and control group proportion = 0.35) as a reference to calculate the sample size which returned the largest one. One hundred twenty-eight teeth (64 cases and 64 controls) were considered necessary. After data collection and collation, a total of 140 individuals were used in this retrospective case-control study.

### 2.2. Inclusion Criteria

The inclusion criteria were as follows: (1) patients with periodontitis who suffered from endo-periodontal lesions, (2) patients who completed treatment for both endodontic and periodontal components, including acceptable quality of nonsurgical root canal treatment and periodontal initial therapy, (3) follow-up maintained over six months, (4) availability of records with all clinical parameters and radiographic examination results, and (5) patients older than 18 years.

### 2.3. Exclusion Criteria

Individuals were excluded based on the following criteria: (1) patients who had root fracture or cracking, root canal, or pulp chamber perforation or external root resorption; (2) patients who underwent periodontal or periapical surgery; (3) patients who had systemic diseases, such as hypertension, diabetes, heart disease, liver disease, or kidney disease; or (4) patients with incomplete medical records.

### 2.4. Diagnostic Criteria

Patients with periodontitis were diagnosed and classified per the AAP case definitions [14]. For teeth with endo-periodontal lesions, the diagnostic criteria were as follows. Major diagnostic criteria are (1) periodontal probing depth (PD) ≥5 mm, (2) clinical attachment level (CAL) ≥3 mm, and (3) patients being aware of pulp symptoms, including spontaneous pain history or having negative or altered pulp vitality tests. Minor diagnostic criteria are (1) red and swollen gums, (2) bleeding on probing, (3) radiographic examination revealing varying degrees of alveolar bone resorption, (4) occlusal discomfort, (5) tooth mobility (TM), (6) discoloration due to pulp necrosis, (7) purulent exudate, and (8) sinus tract [15–17].

### 2.5. endo-periodontal Lesions Classification

A new classification was adopted after the 2017 world workshop on the classification of periodontal and peri-implant diseases and conditions. The consensus report divided endo-periodontal classifications into endo-periodontal lesions with or without root damage. Endo-periodontal lesions without root damage were further divided into endo-periodontal lesions in patients with periodontitis versus patients without periodontitis. Each category included three grades. All teeth selected in this study were from patients with grade 2-3 endo-periodontal lesions [18].

### 2.6. Demographic Information Collection, Clinical Measurements, and Radiographic Assessments

Demographic data, including age, gender and smoking history, clinical and radiographic parameters, and clinical symptoms, including whether the patient experienced occlusal discomfort, swollen gums, or history of spontaneous pain, were collected from the medical records. Patients were divided into two categories based on their smoking habits (current smokers or nonsmokers, including former smokers) [19]. Smokers were defined as those who continued their smoking habit at diagnosis or had quit smoking less than 5 years ago. Nonsmokers were defined as those who had never smoked or were former smokers who had quit smoking at least 5 years prior [20].

The patients' clinical parameters including PD, CAL, sulcus bleeding index (SBI, scored from 0 to 5) [21], simplified oral hygiene index (OHI-S, scored from 0 to 6) [22], TM (scored from I to III) [23], and alveolar resorption (horizontal and angular resorption) [24] of the involved teeth were recorded at baseline, before periodontal therapy and 6 months after periodontal therapy. Horizontal resorption was defined as sites showing an even radiographic appearance of an interdental alveolar ridge paralleling an imaginary line between the cementoenamel junctions of adjacent teeth or having an intrabony component <2 mm [24]. Angular resorption was assigned to sites revealing radiographic signs of increased bone resorption at either the mesial or distal aspect of an interdental alveolar ridge and had the bottom of the oblique radiolucency ≥2 mm apical to the most coronal level of the interproximal alveolar bone [24]. The full-mouth values of the PD, CAL, and SBI were recorded to determine the full-mouth periodontitis severities (mild, moderate, or severe) [14]. The clinical periodontal parameters (PD, CAL, and SBI) of each tooth were assessed at six sites per tooth (mesiobuccal, distobuccal, mid-buccal, mesiolingual, distolingual, and mid-lingual) using a manual periodontal probe (UNC-15, Hu-Friedy, Chicago, IL, USA).

Intraoral periapical radiographs were taken at baseline, before periodontal therapy and 6 months after periodontal therapy using an intraoral X-ray machine (PLANMECA, Helsinki, Finland) with an exposure of 10 mA and 70 kVp. A parallel technique was used in the radiographic examinations to ensure reproducible angles using the Rinn XCP Dental X-ray Positioning device (Dentsply International Inc., Philadelphia, Pennsylvania, USA). The results were analyzed using visualization software (Planmeca Romexis 5.0, Helsinki, Finland). The clinical crown-root ratio (CR) was calculated, and the periapical index (PAI, scored from 1 to 4) [25] and alveolar resorption type (horizontal and angular resorption) were measured from the radiographic examinations. The number of root canals of the involved teeth was obtained via periapical radiographs and was determined during the endodontic treatment.

All clinical and radiographic examinations were measured independently by two examiners (S. W. Y. and P. C. L.) who were unaware of the status. These examiners had been trained using the same standard and could achieve consistent examination results. The *k* coefficients, or intraclass correlation coefficients between examiners, ranged from 0.83 to 0.92 for the PD evaluation, from 0.77 to 0.85 for CAL, from 0.90 to 0.95 for SBI, from 0.95 to 0.98 for TM, from 0.78 to 0. 85 for CR, and from 0.88 to 0.94 for PAI.

### 2.7. Therapeutic Procedures

Patients underwent clinical and radiographic examinations at baseline. The root canal treatment was started and performed over two visits. The tooth involved was isolated with a rubber dam before the root canal treatment was performed. The coronal portion of the root canal was removed using drills (DENTSPLY Maillefer, Tulsa, Oklahoma), and the working length was determined with an electronic apex locator (Dentsply International Inc., Philadelphia, Pennsylvania) and then confirmed radiographically. Root canals were shaped with the crown-down technique using rotary nickel-titanium instruments (Dentsply International Inc., Philadelphia, Pennsylvania). Solutions of 3% sodium hypochlorite (NaOCl; Sainsbury plc, London, UK) and 17% EDTA (Prevest Denpro Ltd, Jammu, India) were used as root canal irrigants. Root canals were dried with paper points (Dentsply International Inc., Philadelphia, Pennsylvania) and were then filled with calcium hydroxide paste (Ivoclar Vivadent AG, Schaan, Principality of Liechtenstein). Intermediate restorative material (Dentsply Ltd., Weybridge, UK) was used for a temporary filling. Patients were recalled to finish the final root canal treatment after approximately 7 days. The paste was removed with drills (DENTSPLY Maillefer, Tulsa, Oklahoma), and 3% sodium hypochlorite and 17% EDTA were used as root canal irrigants. Root canals were dried with paper points and were obturated with gutta-percha (Dentsply International Inc., Philadelphia, Pennsylvania) and zinc oxide-eugenol using vertical compaction technique. The effect of root canal filling was confirmed by inspecting the canals under an intraoral X-ray machine (PLANMECA, Helsinki, Finland). Finally, flowable resin composite (3M ESPE, St Paul, Minnesota) was used to seal the canal orifices by at least 2 mm depth. The remaining coronal restoration was made with composite resin (3M ESPE, St Paul, Minnesota). When retention of composite resin was insufficient, a fibre post (3M ESPE, St Paul, Minnesota) was bonded into the canal space. Subsequently, full-mouth supragingival scaling was performed after 1-2 months using an ultrasonic scaler (Suprasson P5 Booster; Satelec, Merignac Cedex, France), and subgingival scaling and root planning were performed over two visits at a one-week interval [26, 27]. Patients were recalled to the clinic and reevaluated before periodontal therapy (1 to 2 months after root canal therapy) and 6 months after periodontal therapy. Oral hygiene instructions were reinforced to patients at each visit, including how to brush their teeth and how to use dental floss and interdental toothbrushes. Professional supragingival oral prophylaxis was also administered. The curative effect was evaluated based on patients' clinical symptoms and parameters at 6 months [16, 28, 29].

### 2.8. Group Standard

Compared with the baseline and based on patients' clinical symptoms and parameters 6 months after the combined periodontal-endodontic treatment, we divided these teeth into high and low responder groups [30, 31]:

High responder group: no discomfort, masticatory function being improved, clinical examination showing no redness or swollen gums or mild gingival inflammation, PD ≤ 5 mm or at least 2 mm reduction in probing pocket depth, SBI ≤ 2, descending and invariable CAL and TM, no sinus tract, no pain, and no increased PAI.

Low responder group: disease progression, patients having self-conscious symptoms, or the chewing function being impaired, clinical examination showing redness and swollen gums, PD > 5 mm or less than 2 mm reduction in probing pocket depth, SBI ≥ 3, increased CAL or TM, sinus tract present, pain (+), or increased PAI.

### 2.9. Statistical Analysis

All analyses were performed using statistical software (SPSS, version 21.0, IBM, Armonk, New York, USA). Demographic data and the radiographic and clinical parameters are expressed as the means (±SD) or frequency distributions. The independent-samples *t*-test, the paired-samples *t*-test, and the Mann–Whitney *U* test were applied for continuous variables, including age, PD, CAL, OHI-S, and CR, and the chi-square and ridit tests were used for categorical variables, including gender, smoking, SBI, TM, PAI, full-mouth periodontitis severities, discomfort when chewing, alveolar resorption type, and number of root canals. The values that were statistically significant at the univariable level in Tables 1 and 2 and were associated with the clinical significance were analyzed via logistic regression analysis, and differences were considered significant at *P* < 0.05. All significance tests were two-tailed.

## 3. Results

The clinical parameters and symptoms were compared at baseline, before periodontal therapy (1 to 2 months after root canal therapy) and 6 months after periodontal therapy (Table 3). Compared with those at baseline, the values of TM (*P* = 0.036), PAI (*P* = 0.002), and discomfort when chewing (*P* = 0.013) were changed significantly after root canal therapy, and the values of PD (*P* < 0.01), CAL (*P* < 0.01), SBI (*P* < 0.01), TM (*P* = 0.004), OHI-S (*P* = 0.016), full-mouth periodontitis severity (*P* = 0.031), PAI (*P* = 0.002), and discomfort when chewing (*P* = 0.002) were improved significantly in 6 months after periodontal therapy. Compared with those after root canal therapy, the values of PD (*P* < 0.01), CAL (*P* < 0.01), SBI (*P* < 0.01), TM (*P* = 0.002), OHI-S (*P* = 0.009), full-mouth periodontitis severity (*P* = 0.031), PAI (*P* = 0.042), and discomfort when chewing (*P* = 0.009) were significantly different from those at 6 months after periodontal therapy (*P* < 0.05, Table 3).

All the teeth were divided into high and low responder groups based on curative effect. The high responder group included 72 teeth, with an average patient age of 49.58 ± 11.12, with 44 teeth from male patients, 28 teeth from female patients, and a male-to-female ratio of 1.57 : 1. The low responder group included 68 teeth, with an average patient age of 48.00 ± 10.83, with 38 teeth from male patients, 30 teeth from female patients, and a male-to-female ratio of 1.27 : 1. No significant differences were found in age or gender between the high and low responder groups (*P* < 0.05). The proportions of smoking to nonsmoking participants in the high and low responder groups were 11 : 25 and 20 : 14, respectively. The proportion of smokers in the low responder group was significantly higher than that in the high responder group (*P* < 0.05, Table 1).

Table 2 shows the clinical parameters of the high and low responder groups at baseline. The univariate analysis results revealed significant differences in PD (*P* < 0.001), CAL (*P* < 0.001), TM (*P* = 0.004), full-mouth periodontitis severity (*P* = 0.004), CR (*P* = 0.022), PAI (*P* = 0.002), and the number of root canals (*P* < 0.001) between the high and low responder groups. In contrast, no significant differences were found in SBI (*P* = 0.080), OHI-S (*P* = 0.620), or alveolar resorption type (*P* = 0.089) of the involved teeth between the high and low responder groups (*P* < 0.05, Table 2).

To further explore factors affecting endo-periodontal lesion prognosis, logistic regression analysis was conducted on the statistically significant independent variables based on the results of univariate analysis results presented in Tables 1 and 2 and the clinical significance, including smoking, PD, CAL, TM, PAI, CR, full-mouth periodontitis severity, and number of root canals. Smoking (odds ratio (OR) = 5.18, 95% confidence interval (CI) = 1.14 to 23.63, *P* = 0.034), PD (OR = 2.41, 95% CI = 1.24 to 4.65, *P* = 0.009), CAL (OR = 2.34, 95% CI = 1.30 to 4.22, *P* = 0.005), full-mouth periodontitis severity (OR = 2.84, 95% CI = 1.07 to 7.52, *P* = 0.036), and number of root canals (OR = 2.91, 95% CI = 1.22 to 6.94, *P* = 0.016) were significantly associated with the endo-periodontal lesion prognosis (*P* < 0.05, Table 4).

## 4. Discussion

The results of this study showed that only the parameters TM, PAI, and discomfort when chewing were improved after root canal therapy, which suggested that only relying on root canal therapy cannot completely cure grade 2-3 endo-periodontal lesions in patients with periodontitis. This kind of endo-periodontal lesions usually requires both root canal therapy and periodontal therapy to eliminate both endodontic and periodontal microorganisms [3, 17]. Therefore, a proper periodontal therapy will be necessary if we want to improve teeth with grade 2-3 endo-periodontal lesions in patients with periodontitis [3].

Methods for studying prognostic factors include cohort studies, randomized controlled trials, and retrospective studies. In this study, we performed a retrospective case-control study. The exposure data of the case-control study are from the records at baseline and collected before grouping. If the results of the study showed that exposure factors were associated with the disease or the outcomes, then the association was in accordance with the chronological order of causal inference. Moreover, the recall bias is small or could possibly be avoided; therefore, the inference of causality could be more powerful.

In this study, we focused on the prognosis of endo-periodontal lesions instead of their morbidity; therefore, we split the patients into high and low responder groups according to the clinical parameters at the 6-month follow-up and then analyzed the baseline factors via univariate and multivariate analyses to identify the prognostic factors of endo-periodontal lesions treated nonsurgically and to provide guidance for clinicians to make prognostic judgements regarding endo-periodontal lesions. Finally, smoking, PD, CAL, severities of full-mouth periodontitis at baseline, and number of root canals were found to be prognostic factors of endo-periodontal lesions treated nonsurgically.

Smoking impacts the prognosis of both periodontal and endodontic diseases. Hyman and Reid [32] reported that smokers have a higher risk of developing chronic periodontitis than do nonsmokers. Recently, Bunaes et al. [33] found that smokers respond less favorably to periodontal therapy than do nonsmokers. Three reasons explain why smoking negatively affects patients' periodontitis prognosis. First, smoking negatively affects periodontal tissue by modifying the immune response, the healing capacity of the periodontium, and the microbiota composition [34–37]. Second, periodontal tissue is rich in blood vessels, but the nicotine in cigarettes constricts the blood vessels, affecting circulation around the periodontal tissue and inhibiting its metabolism [38]. Third, smoking affects the differentiation and attachment of periodontal fibroblasts as well as osteoblast activity, which can inhibit new alveolar bone attachment formation and regeneration [39, 40]. Moreover, smoking can increase the risk of losing endodontically treated teeth [19] and has been reported as a significant prognostic factor for developing apical periodontitis [41]. However, some studies revealed that smoking was not significantly associated with apical periodontitis after adjustment for age, marginal bone level, and quality of root canal filling [42, 43]. The influence of smoking on apical periodontitis still requires further investigation [44].

The PD and CAL values of the involved teeth reflect the periodontitis severity. The initial periodontal therapeutic prognosis will be poorer in teeth with more severe periodontitis. The periodontal status of involved teeth can also be related to the prognosis of the pulp inflammation. Khalighinejad et al. [19] performed a 9-year retrospective investigation and found that the periodontal status of the involved teeth was a prognostic factor for nonsurgical root canal treatment.

The results of this study demonstrate that the severity of full-mouth periodontitis is a prognostic factor for endo-periodontal lesions treated nonsurgically. The full-mouth periodontal status has been related to root canal treatment outcomes. Ruiz et al. [45] reported that the risk of developing apical periodontitis in endodontically treated teeth is 5.19 times higher for patients with periodontal disease than for patients without periodontal disease. Severe periodontitis with adequate periodontal treatment exposes dentinal tubules to the oral environment, which could serve as an entry point for bacteria from the periodontium to the root canal system, potentially impacting the prognosis of apical periodontitis in endodontically treated teeth [45–47].

Notably, the number of root canals differed significantly between the high and low responder groups based on logistic regression analysis results. Root canals of multirooted teeth are finer than those of single-rooted teeth, which increases the difficulty of root canal treatment. Root furcation and complex pathways between root canals make it challenging to eliminate periodontal and endodontic inflammation. Although the prognosis of endo-periodontal lesions treated nonsurgically is worse in multirooted teeth than that in single-rooted teeth, the clinical survival rate of multirooted teeth is higher than that of single-rooted teeth because not all roots in multirooted teeth suffer from the same loss of supporting tissue, and some surgeries, such as root resection and root separation, can preserve the involved multirooted teeth [48].

Accurately assessing the status of the involved tooth pulp was important in this study. We recruited teeth with unambiguous pulp inflammation symptoms, including histories of spontaneous pain, negative or altered responses to pulp vitality tests, tooth discoloration due to pulp necrosis, pain on palpation or percussion, or sinus tract. Furthermore, the pulp's status was reassessed during the root canal treatment. Combining the history and the clinical and radiographic examination results was considered to reveal the presence of an infected root canal system, indicating that root canal treatment was the mandatory treatment.

To our knowledge, this is the first study of the prognostic factors of grade 2-3 endo-periodontal lesions treated nonsurgically in patients with periodontitis designed as a case-control study and using a multivariable model. The results showed that smoking and periodontal conditions, such as PD, CAL, and full-mouth periodontitis severities, were associated with the prognosis of the involved teeth, whereas the endodontic conditions, such as PAI, did not show this association. These results may help guide clinicians in determining the endo-periodontal lesion prognosis and developing targeted treatments in accordance with the patient's wishes. However, there are some limitations to this study. First, the direct evidence of bacterial correlation in endo-periodontal lesions is absent. Though we did not provide evidence via bacterial detection, all the teeth we collected had obvious symptoms of both periodontitis and pulpitis; periodontitis was diagnosed according to the AAP case definitions; pulpitis was determined by the symptoms and clinical examinations and included spontaneous pain history, PAI, sinus tract, or having negative or altered pulp vitality tests. In this way, some endo-periodontal lesions without obvious symptoms of pulpitis may not be collected, but all the teeth we recruited in this study had to have endo-periodontal lesions. Second, this study used conventional radiography, but using CBCT imaging would be more advantageous to help clinicians comprehensively observe the bone resorption of the involved teeth and determine the number of root canals. In addition, further well-designed studies, such as randomized control trials, are required to substantiate the conclusion of the present study.

## 5. Conclusion

From this 6-month follow-up retrospective study, we concluded that the factors affecting the prognosis of nonsurgical treatment of grade 2-3 endo-periodontal lesions in patients with periodontitis include smoking, PD, CAL, full-mouth periodontitis severity, and the number of root canals.

## Figures and Tables

**Table 1:** Demographic data of the patients in the high and low responder groups at baseline.

Demographic data	High responder (*n* = 72)	Low responder (*n* = 68)	*P* value
Gender^*∗∗*^			0.657
Male	44 (61%)	38 (56%)	
Female	28 (39%)	30 (44%)	
Age (years, *x* ± *s*)^†^	49.58 ± 11.12	48.00 ± 10.83	0.991
Smoking^*∗∗*^			0.032^‡^
No	50 (70%)	28 (44%)	
Yes	22 (30%)	40 (56%)	

^*∗*^Differences between the two groups were analyzed using the chi-square test. ^†^Differences between the two groups were analyzed using the independent-samples *t*-test. ^‡^*P* < 0.05.

**Table 2:** Clinical parameters of the teeth in the high and low responder groups at baseline.

Clinical parameters	High responder (*n* = 72)	Low responder (*n* = 68)	*P* value
PD (mm, *x* ± *s*)^*∗*^	5.78 ± 1.31	7.18 ± 1.49	<0.001^#^
CAL (mm, *x* ± *s*)^*∗*^	5.42 ± 1.52	7.35 ± 1.79	<0.001^#^
SBI^†^			0.080
1	10 (14%)	6 (9%)	
2	20 (28%)	12 (18%)	
3	26 (36%)	20 (29%)	
4	16 (22%)	30 (44%)	
TM^†^			0.004^#^
0	12 (17%)	2 (3%)	
I	22 (30%)	12 (17%)	
II	34 (47%)	40 (59%)	
III	4 (6%)	14 (21%)	
OHI-S (*x* ± *s*)^*∗*^	2.40 ± 0.78	2.51 ± 0.36	0.620
Full-mouth periodontitis severity^†^			0.004^#^
1	26 (36%)	8 (12%)	
2	36 (50%)	26 (38%)	
3	10 (14%)	34 (50%)	
Alveolar resorption type^§^			0.089
Horizontal resorption	40 (56%)	24 (35%)	
Vertical resorption	32 (44%)	44 (65%)	
CR (*x* ± *s*)^*∗*^	0.67 ± 0.08	0.73 ± 0.12	0.022^¶^
PAI^†^			0.002^#^
1	22 (30%)	6 (9%)	
2	30 (42%)	22 (32%)	
3	18 (25%)	30 (44%)	
4	2 (3%)	10 (15%)	
Number of root canals^‡^			<0.001^#^
1	28 (39%)	12 (18%)	
2	30 (41%)	12 (18%)	
3	12 (17%)	30 (44%)	
4	2 (3%)	14 (20%)	

^*∗*^Differences between the two groups were analyzed using the independent-sample *t*-test. ^†^Differences between the two groups were analyzed using the ridit test. ^‡^Differences between the two groups were analyzed using the Mann–Whitney *U* test. ^§^Differences between the two groups were analyzed using the chi-square test. ^¶^*P* < 0.05. ^#^*P* < 0.01.

**Table 3:** Changes in clinical parameters at baseline, before periodontal therapy, and 6 months after periodontal therapy.

Clinical parameters	Baseline (*n* = 140)	Before periodontal therapy (*n* = 140)	6 months after periodontal therapy (*n* = 140)
PD (mm, *x* ± *s*)^*∗∗*^	6.46 ± 1.55	6.12 ± 1.51	5.03 ± 1.49^Aa^
CAL (mm, *x* ± *s*)^*∗∗*^	6.35 ± 1.62	6.05 ± 1.86	4.57 ± 1.79^Aa^
SBI^†^			
0	0 (0%)	0 (0%)	12 (9%)^Aa^
1	16 (11%)	15 (11%)	38 (27%)
2	32 (23%)	35 (25%)	38 (27%)
3	46 (33%)	50 (36%)	30 (21%)
4	46 (33%)	40 (28%)	22 (16%)
TM^†^			
0	14 (10%)	16 (11%)^A^	24 (3%)^Aa^
I	32 (23%)	30 (22%)	40 (17%)
II	78 (56%)	80 (57%)	66 (59%)
III	16 (11%)	14 (10%)	10 (21%)
OHI-S (*x* ± *s*)^*∗∗*^	2.38 ± 0.48	2.35 ± 0.39	0.89 ± 0.47^Aa^
Full-mouth periodontitis severity^†^			
1	34 (24%)	34 (24%)	50 (36%)^Aa^
2	62 (44%)	62 (44%)	56 (40%)
3	44 (32%)	44 (33%)	34 (24%)
Alveolar resorption type^‡^			
Horizontal resorption	64 (46%)	65 (46%)	66 (47%)
Vertical resorption	76 (54%)	75 (54%)	74 (53%)
CR ((*x* ± *s*)^*∗*^)^*∗*^	0.70 ± 0.11	0.72 ± 0.15	0.68 ± 0.12
PAI^†^			
0	0 (0%)	22 (16%)^A^	28 (20%)^Aa^
1	28 (20%)	38 (27%)	44 (31%)
2	52 (37%)	40 (29%)	39 (28%)
3	48 (34%)	30 (21%)	22 (16%)
4	12 (9%)	10 (7%)	7 (5%)
Discomfort when chewing^‡^			
No	27 (20%)	72 (51%)^A^	109 (78%)^Aa^
Yes	113 (80%)	68 (49%)	31 (22%)

^*∗*^Differences between the two groups were analyzed using the paired-sample *t*-test. ^†^Differences between the two groups were analyzed using the ridit test. ^‡^Differences between the two groups were analyzed using the chi-square test. ^A^*P* < 0.05 compared to baseline. ^a^*P* < 0.05 compared to before periodontal therapy.

**Table 4:** Logistic regression analyses for prognostic factors of grade 2-3 endo-periodontal lesions treated nonsurgically in patients with periodontitis.

Clinical parameters	*Β*	Standard error	OR (95% CI)	*P* value
PD	0.877	0.337	2.41 (1.24 to 4.65)	0.009^†^
CAL	0.849	0.301	2.34 (1.30 to 4.22)	0.005^†^
Smoking	1.645	0.775	5.18 (1.14 to 23.63)	0.034^*∗*^
Full-mouth periodontitis severity	1.043	0.497	2.84 (1.07 to 7.52)	0.036^*∗*^
Number of root canals	1.069	0.443	2.91 (1.22 to 6.94)	0.016^*∗*^

^*∗*^ *P* < 0.05. ^†^*P* < 0.01.

## Data Availability

The data used to support the findings of this study are included within the article.
